# An Overview of Bayesian Methods for Neural Spike Train Analysis

**DOI:** 10.1155/2013/251905

**Published:** 2013-11-17

**Authors:** Zhe Chen

**Affiliations:** ^1^Department of Brain and Cognitive Sciences, Massachusetts Institute of Technology, 43 Vassar Street, Cambridge, MA 02139, USA; ^2^Picower Institute for Learning and Memory, Massachusetts Institute of Technology, Cambridge, MA 02139, USA

## Abstract

Neural spike train analysis is an important task in computational neuroscience which aims to understand neural mechanisms and gain insights into neural circuits. With the advancement of multielectrode recording and imaging technologies, it has become increasingly demanding to develop statistical tools for analyzing large neuronal ensemble spike activity. Here we present a tutorial overview of Bayesian methods and their representative applications in neural spike train analysis, at both single neuron and population levels. On the theoretical side, we focus on various approximate Bayesian inference techniques as applied to latent state and parameter estimation. On the application side, the topics include spike sorting, tuning curve estimation, neural encoding and decoding, deconvolution of spike trains from calcium imaging signals, and inference of neuronal functional connectivity and synchrony. Some research challenges and opportunities for neural spike train analysis are discussed.

## 1. Introduction

Neuronal action potentials or spikes are the basic language that neurons use to represent and transmit information. Understanding neuronal representations of spike trains remains a fundamental task in computational neuroscience [1, 2]. With the advancement of multielectrode array and imaging technologies, neuroscientists have been able to record a large population of neurons at a fine temporal and spatial resolution [3]. To extract (“read out”) information from or inject/restore (“write in”) signals to neural circuits [4], there are emerging needs for modeling and analyzing neural spike trains recorded directly or extracted indirectly from neural signals, as well as building closed-loop brain-machine interfaces (BMIs). Many good examples and applications can be found in the volumes of the current or other special issues [5, 6].

In recent years, cutting-edge Bayesian methods have gained increasing attention in the analysis of neural data and neural spike trains. Despite its well-established theoretic principle since the inception of Bayes' rule [7], Bayesian machinery has not been widely used in large-scaled data analysis until very recently. This was partially ascribed to two facts: first, the development of new methodologies and effective algorithms; second, the ever-increasing computing power. The major theoretic or methodological development has been reported in the field of statistics, and numerous algorithms were developed in applied statistics and machine learning for successful real-world applications [8]. It is time to push this research frontier to neural data analysis. With this purpose in mind, this paper provides a tutorial review on the basic theory and the state-of-the-art Bayesian methods for neural spike train analysis.

The rest of the paper is organized as follows.  Section 2 presents the background information about statistical inference and estimation, Bayes' theory, and statistical characterization of neural spike trains.  Section 3 reviews several important Bayesian modeling and inference methods in light of different approximation techniques.  Section 4 reviews a few representative applications of Bayesian methods for neural spike train analysis. Finally, Section 5 concludes the paper with discussions on a few challenging research topics in neural spike train analysis.

## 2. Background

### 2.1. Estimation and Inference: Statistic versus Dynamic

Throughout this paper, we denote by *Y* the observed variables, by *X* the hidden variables and by *θ* an unknown parameter vector, and by ^*⊤*^ the transpose operator for vector or matrix. We assume that *p*(*Y* | *X*, *θ*) has a regular and well-defined form of the likelihood function. For neural spike train analysis, *Y* typically consists of time series of single or multiple spike trains. Given a fixed time interval (0, *T*], by time discretization we have *Y* = {*Y*_1_, *Y*_2_,…, *Y*_*K*_} (where *K* = *T*/Δ and Δ denotes the temporal bin size). A general statistical inference problem is stated as follows: given observations *Y*, estimate the unknown hidden variable *X* with a known *θ*, or estimate *θ* alone, or jointly estimate *θ* and *X*. The unknown variables *θ* and *X* can be either static or dynamic (e.g., time-varying with a Markovian structure). We will review the approaches that tackle these scenarios in this paper.

There are two fundamental approaches to solve the inference problem: likelihood approach and Bayesian approach. The likelihood approach [9] computes a point estimate by maximizing the likelihood function and represents the uncertainty of estimate via confidence intervals. The maximum likelihood estimate (m.l.e.) is asymptotically consistent, normal, and efficient, and it is invariant to reparameterization (i.e., functional invariance). However, the m.l.e. is known to suffer from overfitting, and therefore model selection is required in statistical data analysis. In contrast, the Bayesian philosophy lets data speak for themselves and models the unknowns (parameters, latent variables, and missing data) and uncertainties (which are not necessarily random) with probabilities or probability densities. The Bayesian approach computes the full posterior of the unknowns based on the rules of probability theory; the Bayesian approach can resolve the overfitting problem in a principled way [7, 8].

### 2.2. Bayesian Inference

The foundation of Bayesian inference is given by Bayes' rule, which consists of two rules: *product rule* and *sum rule*. Bayes' rule provides a way to compute the conditional, joint, and marginal probabilities. Specifically, let *X* and *Y* be two continuous random variables (r.v.); the conditional probability *p*(*X* | *Y*) is given by

(1) $$\begin{array}{c} p\left(X\;|\;Y\right)=\frac{p\left(X,Y\right)}{p\left(Y\right)}=\frac{p\left(Y\;|\;X\right)p\left(X\right)}{\int p\left(Y\;|\;X\right)p\left(X\right)dX}. \end{array}$$

If *X* = {*X*_*i*_} is discrete, then (1) is rewritten as

(2) $$\begin{array}{c} p\left(X_{i}\;|\;Y\right)=\frac{p\left(X_{i},Y\right)}{p\left(Y\right)}=\frac{p\left(Y\;|\;X_{i}\right)p\left(X_{i}\right)}{\sum_{j}p\left(Y\;|\;X_{j}\right)p\left(X_{j}\right)}. \end{array}$$

In Bayesian language, *p*(*Y* | *X*), *p*(*X*), and *p*(*X* | *Y*) are referred to as the likelihood, prior and posterior, respectively. The Bayesian machinery consists of three types of basic operations: *normalization, marginalization,* and *expectation*, all of which involve integration. And the optimization problem consists in maximizing the posterior *p*(*X* | *Y*) and finding the maximum a posteriori (MAP) estimate *X*_MAP_ = arg_*X*_max⁡*p*(*X* | *Y*). Notably, except for very few scenarios (i.e., Gaussianity), most integrations are computationally intractable or costly when dealing with high-dimensional problems. Therefore, for the sake of computational tractability, various types of approximations are often assumed at different stages of the inference procedure.

More specifically, for the state and parameter estimation problem, Bayesian inference aims to infer the joint posterior of the state and the parameter using Bayes' rule

(3) p(X,θ ∣ Y)≈p(X ∣ Y)p(θ ∣ Y)=p(Y ∣ X,θ)p(X)p(θ)p(Y)=p(Y ∣ X,θ)p(X)p(θ)∬p(Y ∣ X,θ)p(X)p(θ)dX dθ,

where the first equation assumes a factorial form of the posterior for the state and the parameter (first-stage approximation) and *p*(*X*) and *p*(*θ*) denote the prior distributions for the state and parameter, respectively. The denominator of (3) is a normalizing constant known as the partition function. When dealing with a prediction problem for unseen data Y*, we compute the posterior predictive distribution

(4) $$\begin{array}{c} p\left(Y^{*}\;|\;Y\right)=\iint p\left(Y^{*}\;|\;Y,\theta ,X\right)p\left(X\;|\;Y\right)p\left(\theta \;|\;Y\right)dX\;d\theta \end{array}$$

or its expected mean ${\hat{Y}}^{*}={𝔼}_{p(Y^{*}|Y)}[Y^{*}]=∭Y^{*}p\left(Y^{*}|Y,\theta ,X\right)p\left(X|Y\right)p\left(\theta |Y\right)dX\;d\theta \;dY^{*}$.

Sometimes, instead of maximizing the posterior, Bayesian inference attempts to maximize the marginal likelihood (also known as “evidence”) *p*(*Y*) as follows:

(5) $$\begin{array}{c} p\left(Y\right)=\iint p\left(Y\;|\;X,\theta \right)p\left(X\right)p\left(\theta \right)dX\;d\theta . \end{array}$$

The second-stage approximation in approximate Bayesian inference deals with the integration in computing (3), (4), or (5), which will be reviewed in Section 3.

*Note.* Maximum likelihood inference can be viewed as a special case of Bayesian inference, in which *θ* is represented by a Dirac-delta function centered at the point estimate ${\hat{\theta }}_{\text{m}.\text{l}.\text{e}.}$; namely, $p(\theta )=\delta (\theta -{\hat{\theta }}_{\text{m}.\text{l}.\text{e}.})$. Nevertheless, Bayesian inference can still be embedded into likelihood inference to estimate *p*(*X*) given a point estimate of *θ*. The *p*(*X*) can either have an analytic form (with finite natural parameters) or be represented by Monte Carlo samples; the latter approach may be viewed as a specific case of Monte Carlo expectation-maximization (EM) methods.

### 2.3. Characterization of Neural Spike Trains

Neural spike trains can be modeled as a simple (temporal) point process [10]. For a single neural spike train observed in (0, *T*], we often discretize it with a small bin size Δ such that each bin contains no more than one spike. The conditional intensity function (CIF), denoted as *λ*(*t* | *H*_*t*_), is used to characterize the spiking probability of a neural point process as follows:

(6) $$\begin{array}{c} \lambda \left(t\;|\;H_{t}\right)=\underset{\Delta \to 0}{\lim}\frac{\text{Pr}\left\{\text{spike}\;\;\text{in}\left(t,t+\Delta \right]\;|\;H_{t}\right\}}{\Delta }, \end{array}$$

where *H*_*t*_ denotes all history information available up to time *t* (that may include spike history, stimulus covariate, etc.). When *λ*(*t* | *H*_*t*_) is history independent, then the stochastic process is an inhomogeneous Poisson process. For notation simplicity, we sometimes use *λ*_*t*_ to replace *λ*(*t* | *H*_*t*_) when no confusion occurs. When Δ is sufficiently small, the product *λ*(*t* | *H*_*t*_)Δ is approximately equal to the probability of observing a spike within the interval ((*t* − 1)Δ, *t*Δ]. Assuming that the CIF *λ*_*t*_ is characterized by a parameter *θ* and an observed or latent variable *X*, then the point process likelihood function is given as [11–13]

(7) p(Y ∣ X,θ)=exp⁡{∫0Tlog⁡⁡λ(τ ∣ θ,X)dy(τ)−∫0Tλ(τ ∣ θ,X)dτ},

where *dy*(*t*) is an indicator function of the spike presence within the interval ((*t* − 1)Δ, *t*Δ]. In the presence of multiple spike trains from *C* neurons, assuming that multivariate point process observations are conditionally independent at any time *t* given a new parameter *θ*, one then has

(8) p(Y1:C ∣ X,θ)=∏c=1Cp(Yc ∣ X,θ)=∏c=1Cexp⁡{∫0Tlog⁡⁡λc(τ ∣ θ,X)dyc(τ)−∫0Tλc(τ ∣ θ,X) dτ}.

Since neural spike trains are fully characterized by the CIF, the modeling goal is then turned to model the CIF, which can have a parametric or nonparametric form. Identifying the CIF and its associated parameters is essentially a neural encoding problem (Section 4.2). A convenient modeling framework is the generalized linear model (GLM) [14, 15], which can model the binary (0/1) or spike count measurements. Within the exponential family, one can use the logit link function to model the binomial distribution, which has a generic form of log⁡(*p*_*t*_/(1 − *p*_*t*_)) = *θ* ^*⊤*^ *X*; one can also use the log link function to model the Poisson distribution, which has a generic form of log⁡(*λ*_*t*_) = *θ* ^*⊤*^ *X*.

In addition, researchers have used the negative binomial distribution to model spike count observations to capture the overdispersion phenomenon (where the variance is greater than the mean statistic). In many cases, for the purpose of computational tractability, researchers often use a Gaussian approximation for Poisson spike counts through a variance stabilization transformation.  Table 1 lists a few population probability distributions for modeling spike count observations.

Another popular statistical model for characterizing population spike trains is the maximum entropy (MaxEnt) model with a log-linear form [16, 17]. Given an ensemble of *C* neurons, the ensemble spike activity can be characterized by the following form:

(9) p(X)=1𝒵(X)exp⁡(∑i=1Cθc〈xc〉+∑i,jCθij〈xixj〉)≡1𝒵(X)exp⁡(∑i=1C+C2θifi(X)),

where *x*_*i*_ ∈ {−1, +1}, 〈·〉 denotes the sample average, 〈*x*_*c*_〉 denotes the mean firing rate of the *c*th neuron, *f*_*i*_(*X*) denotes a generic function of *X* (where the couplings *θ*_*i*_ have to match the measured expectation values 〈*f*_*i*_(*X*)〉), and *𝒵*(*X*) denotes the partition function. The basic MaxEnt model (9) assumes the stationarity of the data and includes the first- and second-order moment statistics but no stimulus component, but these assumptions can be relaxed to further derive an extended model.

An important issue for characterizing neural spike trains is model selection and the associated goodness-of-fit assessment. For goodness-of-fit assessment of spike train models, the reader is referred to [11, 18]. In addition, standard statistical techniques such as cross-validation, leave-one-out, and the receiver-operating-characteristic (ROC) curve may be considered. The model selection issue can be resolved by the likelihood principle based on well-established criteria (such as the *Bayesian information criterion* or *Akaike's information criterion*) [9, 11] or resolved by the Bayesian principle. Bayesian model selection and variable selection will be reviewed in Section 3.4.

## 3. Bayesian Modeling and Inference Methods

The common strategy of Bayesian modeling is to start with specific prior distributions for the unknowns. The prior distributions are characterized by some hyperparameters, which can be directly optimized or modeled by the second-level hyperpriors. If the prior is conjugate to the likelihood, then the posterior has the same form as the prior [8]. Hierarchical Bayesian modeling characterizes the uncertainties of all unknowns at different levels.

In this section, we will review some, either exact or approximate, Bayesian inference methods. The approximate Bayesian inference methods aim to compute or evaluate the integration by approximation. There are two types of approaches to accomplish this goal: deterministic approximation and stochastic approximation. The deterministic approximation can rely on Gaussian approximation, deterministic sampling (e.g., sigma-point approximation [19, 20]) or variational approximation [21–23]. The stochastic approximation uses Monte Carlo sampling to achieve a point mass representation of the probability distribution. These two approaches have been employed to approximate the likelihood or posterior function in many inference problems, such as model selection, filtering and smoothing, and state and parameter joint estimation. Detailed coverage of these topics can be found in many excellent books (e.g., [24–28]).

### 3.1. Variational Bayes (VB)

VB is based on the idea of variational approximation [21–23] and is also referred to as *ensemble learning* [24]. To avoid overfitting in maximum likelihood estimation, VB aims to maximize the marginal log-likelihood or its lower bound as follows:

(10) log⁡⁡p(Y)=log⁡⁡∫dθ∫dXp(θ)p(X,Y ∣ θ)=log⁡⁡∫dθ∫dXq(X,θ)p(θ)p(X,Y ∣ θ)q(X,θ)≥∫dθ∫dXq(X,θ)log⁡p(θ)p(X,Y ∣ θ)q(X,θ)=〈log⁡⁡p(X,Y,θ)〉q+ℋq(X,θ)≡ℱ(q(X,θ)),

where *p*(*θ*) denotes the parameter prior distribution, *p*(*X*, *Y* | *θ*) defines the complete data likelihood, and *q*(*X*, *θ*) is called the variational posterior distribution which approximates the joint posterior of the unknown state and parameter *p*(*X*, *θ* | *Y*). The term *ℋ*_*q*_ represents the entropy of the variational posterior distribution *q*, and *ℱ*(*q*(*X*, *θ*)) is referred to as the free energy. The lower bound is derived based on the *Jensen's inequality* [29]. Maximizing the free energy *ℱ*(*q*(*X*, *θ*)) is equivalent to minimizing the Kullback-Leibler (KL) divergence [29] between the variational posterior and true posterior (denoted by KL(*q*||*p*)); since the KL divergence is nonnegative, we have *ℱ*(*q*) = log⁡*p*(*Y*) − KL(*q*||*p*) ≤ log⁡*p*(*Y*). The optimization problem in (10) can be resorted to the VB-EM algorithm [23] in a similar fashion as the standard EM algorithm [30].

A common (but not necessary) VB assumption is a factorial form of the posterior *q*(*X*, *θ*) = *q*(*X*)*q*(*θ*), although one can further impose certain structure within the parameter space. In the case of mean-field approximation, we have *q*(*X*, *θ*) = *q*(*X*)∏_*i*_ *q*(*θ*_*i*_). With selected priors *p*(*X*) and *p*(*θ*), one can maximize the free energy by alternatively solving two equations: ∂*ℱ*/∂*X* = 0 and ∂*ℱ*/∂*θ* = 0. Specifically, VB-EM inference can be viewed as a natural extension of the EM algorithm, which consists of the following two steps.

- VB-E step: given the available information of *q*(*θ*), maximize the free energy *ℱ* with respect to the function *q*(*X*) and update the posterior *q*(*X*).
- VB-M step: given the available information of *q*(*X*), maximize the free energy *ℱ* with respect to the function *q*(*θ*) and update the posterior *q*(*θ*). The posterior update will have an analytic form provided that the prior *p*(*θ*) is conjugate to the complete-data likelihood function (the conjugate-exponential family).

These two steps are alternated repeatedly until the VB algorithm reaches the convergence (say, the incremental change of *ℱ* value is below a small threshold). Similar to the iterative EM algorithm, the VB-EM inference has local maxima in optimization. To resolve this issue, one may use multiple random initializations or employ a deterministic annealing procedure [31]. The EM algorithm can be viewed as a variant of the VB algorithm in that the VB-M step replaces the point estimate (i.e., *q*(*θ*) = *δ*(*θ* − *θ*_MAP_)) from the traditional M-step with a full posterior estimate. Another counterpart of the VB-EM is the maximization-expectation (ME) algorithm [32], in which the VB-E step uses the MAP point estimate *q*(*X*) = *δ*(*X* − *X*_MAP_), while the VB-M step updates the full posterior.

It is noted that when the latent variables and parameters are intrinsically coupled or statistically correlated, the mean-field approximation will not be accurate, and consequently the VB estimate will be strongly biased. To alleviate this problem, the latent-space VB (LSVB) method [33, 34] aims to maximize a tighter lower bound of the marginal log-likelihood from (10) as follows:

(11) log⁡⁡p(Y)≥∫dXq(X)log⁡⁡p(X,Y)q(X)=∫dXq(X)log⁡⁡∫dθp(X,Y,θ)p(θ)q(X)≡ℱ(q(X))≥max⁡q(θ) ℱ(q(X)q(θ)).

The reader is referred to [33, 34] for more details and algorithmic implementation.

*Note.* (i) Depending on specific problems, the optimization bound of VB methods may not be tight, which may cause a large estimate bias or underestimated variance [35]. Desirably, a *data-dependent* lower bound is often tighter (such as the one used in Bayesian logistic regression [25]). (ii) It was shown in [36] that the VB method for statistical models with latent variables can be viewed as a special case of local variational approximation, where the log-sum-exp function is used to form the lower bound of the log-likelihood. (iii) The VB-EM inference was originally developed for the probabilistic models in the conjugate-exponential family, but it can be extended to more general models based on approximation [37].

### 3.2. Expectation Propagation (EP)

EP is a message-passing algorithm that allows approximate Bayesian inference for factor graphs (one type of probabilistic graphical model that shows how a function of several variables can be factored into a product of simple functions and can be used to represent a posterior distribution) [38]. For a specific r.v. *X* (either continuous or discrete), the probability distribution *p*(*X*) is represented as a product of factors as follows:

(12) $$\begin{array}{c} p\left(X\right)=\prod_{a}f_{a}\left(X\right). \end{array}$$

The basic idea of EP is to “divide-and-conquer” by approximating the factors one by one as follows:

(13) $$\begin{array}{c} f_{a}\left(X\right)\to {\tilde{f}}_{a}\left(X\right) \end{array}$$

and then use the product of approximated term as the final approximation as follows:

(14) $$\begin{array}{c} q\left(X\right)=\prod_{a}{\tilde{f}}_{a}\left(X\right). \end{array}$$

As a result, EP replaces the global divergence KL(*p*(*X*)||*q*(*X*)) by the local divergence between two product chains as follows:

(15) KL(p(X)||q(X))  =KL(fa(X)∏b≠afb(X)||f~a(X)∏b≠af~b(X))  ≈KL(fa(X)∏b≠af~b(X)||f~a(X)∏b≠af~b(X)).

To minimize (15), the EP inference procedure is planned as follows.

*Step 1.* Use message-passing algorithms to pass messages ${\tilde{f}}_{a}(X)$ between factors.

*Step 2.* Given the received message ${\tilde{f}}_{b}(X)$ for factor *a* (for all *b* ≠ *a*), minimize the local divergence to obtain ${\tilde{f}}_{a}(X)$, and send it to other factors.

*Step 3.* Repeat the procedure until convergence.

*Note.* (i) EP aims to find the closest approximation *q* such that KL(*p*||*q*) is minimized, whereas VB aims to find the variational distribution to minimize KL(*q*||*p*) (note that the KL divergence is asymmetric, and KL(*p*||*q*) and KL(*q*||*p*) have different geometric interpretations [39]). (ii) Unlike the global approximation technique (e.g., moment matching), EP uses a local approximation strategy to minimize a series of local divergence.

### 3.3. Markov Chain Monte Carlo (MCMC)

MCMC methods are referred to as a class of algorithms for drawing random samples from probability distributions by constructing a Markov chain that has the equilibrium distribution as the desired distribution [40]. The designed Markov chain is reversible and has detailed balance. For example, given a transition probability *P*, the detailed balance holds between each pair of state *i* and *j* in the state space if and only if *π*_*i*_ *P*_*ij*_ = *π*_*j*_ *P*_*ji*_ (where *π*_*i*_ = Pr(*X*_*t*−1_ = *i*) and *P*_*ij*_ = Pr(*X*_*t*−1_ = *i*, *X*_*t*_ = *j*)). The appealing use of MCMC methods for Bayesian inference is to numerically calculate high-dimensional integrals based on the samples drawn from the equilibrium distribution [41].

The most common MCMC methods are the random walk algorithms, such as the Metropolis-Hastings (MH) algorithm [42, 43] and Gibbs sampling [44]. The MH algorithm is the simplest yet the most generic MCMC method to generate samples using a random walk and then to accept them with a certain acceptance probability. For example, given a random-walk proposal distribution *g*(*Z* → *Z*′) (which defines a conditional probability of moving state *Z* to *Z*′), the MH acceptance probability *𝒜*(*Z* → *Z*′) is

(16) $$\begin{array}{c} 𝒜\left(Z\to Z^{\prime }\right)=\min\left(1,\frac{p\left(Z^{\prime }\right)g\left(Z^{\prime }\to Z\right)}{p\left(Z\right)g\left(Z\to Z^{\prime }\right)}\right), \end{array}$$

which gives a simple MCMC implementation. Gibbs sampling is another popular MCMC method that requires no parameter tuning. Given a high-dimensional joint distribution *p*(*Z*) = *p*(*z*_1_,…, *z*_*n*_), Gibbs sampler draws samples from the individual conditional distribution *p*(*z*_*i*_ | *Z*_−*i*_) in turn while holding others fixed (where *Z*_−*i*_ denote the *n* − 1 variables in *Z* except for *z*_*i*_).

For high-dimensional sampling problems, the random-walk behavior of the proposal distribution may not be efficient. Imagine that there are two directions (increase or decrease in the likelihood space) for a one-dimensional search; there will be 2^*n*^ search directions in an *n*-dimensional space. On average, it will take about 2^*n*^/*n* steps to hit the exact search direction. Notably, some sophisticated MCMC algorithms employ side information to improve the efficiency of the sampler (i.e., the “mixing” of the Markov chain). Examples of non-random-walk methods include successive overrelaxation, hybrid Monte Carlo, gradient-based Langevin MCMC, and Hessian-based MCMC [24, 45–47].

Many statistical estimation problems (e.g., change point detection, clustering, and segmentation) consist in identifying the unknown number of statistical objects (e.g., change points, clusters, and boundaries), which are categorized as the variable-dimensional statistical inference problem. For this kind of inference problem, the so-called reversible jump MCMC (RJ-MCMC) method has been developed [48], which can be viewed as a variant of MH algorithm that allows proposals to change the dimensionality of the space while satisfying the detailed balance of the Markov chain.

*Note.* As discussed in Section 2.2, since the fundamental operations of Bayesian statistics involve integration, the MCMC methods appear naturally as the most generic techniques for Bayesian inference. On the one hand, the recent decades have witnessed an exponential growth in the MCMC literature for its own theoretic and algorithmic developments. On the other hand, there has been also an increasing trend in applying MCMC methods to neural data analysis, ranging from spike sorting, tuning curve estimation, and neural decoding to functional connectivity analysis, some of which will be briefly reviewed in Section 4.

### 3.4. Bayesian Model Selection and Variable Selection

Statistical model comparison can be carried on by Bayesian inference. From Bayes' rule, the model posterior probability is expressed by

(17) $$\begin{array}{c} p\left({ℳ}_{i}\;|\;D\right)\propto p\left(D\;|\;{ℳ}_{i}\right)p\left({ℳ}_{i}\right). \end{array}$$

Under the assumption of equal model priors, maximizing the model posterior is equivalent to maximizing the model evidence (or marginal likelihood) as follows:

(18) p(D ∣ ℳi)=∫θp(D,θ ∣ ℳi)dθ=∫θp(D ∣ θ,ℳi)p(θ ∣ ℳi)dθ.

The Bayes factor (BF), defined as the ratio of evidence between two models, can be computed as [49]

(19) BF=p(D ∣ ℳ1)p(D ∣ ℳ2)=∫p(D,θ1 ∣ ℳ1)dθ1∫p(D,θ2 ∣ ℳ2)dθ2=∫p(θ1 ∣ ℳ1)p(D ∣ θ1,ℳ1)dθ1∫p(θ2 ∣ ℳ2)p(D ∣ θ2,ℳ2)dθ2.

Specifically, the BF is treated as the Bayesian alternative to *P* values for testing hypotheses (in model selection) and for quantifying the degree the observed data support or conflict with a hypothesis [50]. As discussed previously in Section 3.1, the marginal likelihood may be intractable for a large class of probabilistic models. In practice, the BF is often computed based on numerical approximation, such as the Laplace-Metropolis Estimator [51] or sequential Monte Carlo methods [52]. In addition, for a large sample size, the logarithm of the BF can be roughly approximated by the Bayesian information criterion (BIC) [9], whose computation is much simpler without involving numerical integration.

Bayesian model selection can also be directly implemented via the so-called MCMC model composition (MC^3^). The basic idea of MC^3^ is to simulate a Markov chain {*ℳ*(*t*)} with an equilibrium distribution as *p*(*ℳ*_*i*_ | *D*). For each model *ℳ*, define a neighborhood nbd(*ℳ*) and a transition matrix *q* by setting *q*(*ℳ* → *ℳ*′) = 0 for all *ℳ*′ ∉ nbd(*ℳ*). Draw a new sample *ℳ*′ from *q*(*ℳ* → *ℳ*′) and accept the new sample with a probability

(20) $$\begin{array}{c} \min\left\{1,\frac{p\left({ℳ}^{\prime }\;|\;D\right)}{p\left(ℳ\;|\;D\right)}\right\}. \end{array}$$

Otherwise the chain remains unchanged. Once the Markov chain converges to the equilibrium, one can construct the model posterior based on Monte Carlo samples.

Within a fixed model class, it is often desirable to have a compact or sparse representation of the model to alleviate overfitting. Namely, many coefficients of the model parameters are zeros. A very useful approach for variable selection is the so-called automatic relevance determination (ARD) that encourages sparse Bayesian learning [24, 26, 53]. More specifically, ARD provides a way to infer hyperparameters in hierarchical Bayesian modeling. Given the likelihood *p*(*Y* | *θ*) and the parameter prior *p*(*θ* | *ω*) (where *ω* denotes the hyperparameters), one can assign a hyperprior *p*(*ω* | *η*) for *ω* such that the marginal distribution *p*(*θ*) = ∫*p*(*θ* | *ω*)*p*(*ω*)*dω* is peaked and long-tailed (thereby favoring a sparse solution). The hyperprior *p*(*ω*) can be either identical or different for each element in *θ*. In the most general form, we can write

(21) $$\begin{array}{c} p\left(\theta \right)=\prod_{i}p\left(\theta_{i}\right)=\prod_{i}‍\int p\left(\theta_{i}\;|\;\omega_{i}\right)p\left(\omega_{i}\;|\;\eta_{i}\right)d\omega_{i}. \end{array}$$

The hyperprior parameters *η* = {*η*_*i*_} can be fixed or optimized from data. Upon completing Bayesian inference, the estimated mean and variance statistics of some coefficients *θ*_*i*_ will be close to zero (i.e., with the least relevance) and therefore can be truncated. The ARD principle has been widely used in various statistical models, such as linear regression, GLM, and the relevance vector machine (RVM) [26].

### 3.5. Bayesian Model Averaging (BMA)

BMA is a statistical technique aiming to account for the uncertainty in the model selection process [54]. By averaging many different competing statistical models (e.g., linear or Cox regression and GLM), BMA incorporates model uncertainties into parameter inference and data prediction.

Consider an example of GLM involving choosing independent variables and the link function. Every possible combination of choices defines a different model, say {*ℳ*_0_, *ℳ*_1_,…, *ℳ*_*K*_} (where *ℳ*_0_ denotes the null model). Upon computing *K* Bayes factors BF_10_ = *p*(*D* | *ℳ*_1_)/*p*(*D* | *ℳ*_0_), BF_20_ = *p*(*D* | *ℳ*_2_)/*p*(*D* | *ℳ*_0_),…, and BF_*K*0_ = *p*(*D* | *ℳ*_*K*_)/*p*(*D* | *ℳ*_0_), the posterior probability *p*(*ℳ*_*k*_ | *D*) is computed as [54]

(22) $$\begin{array}{c} p\left({ℳ}_{k}\;|\;D\right)=\frac{\pi_{k}\text{B}{\text{F}}_{k0}}{\sum_{i=0}^{K}\pi_{i}\text{B}{\text{F}}_{i0}}, \end{array}$$

where *π*_*k*_ = *p*(*ℳ*_*k*_)/*p*(*ℳ*_0_) denotes the prior odds for model *ℳ*_*k*_ against *ℳ*_0_. In the case of GLM, the marginal likelihood can be approximated by the Laplace method [55].

### 3.6. Bayesian Filtering: Kalman Filter, Point Process Filter, and Particle Filter

Bayesian filtering aims to infer a filtered or predictive posterior distribution of temporal data in a sequential fashion, which is often cast within the framework of state space model (SSM) [13, 56, 57]. Without loss of generality, let **x**_*t*_ denote the state at discrete time *t* and let **y**_0:*t*_ denote the cumulative observations up to time *t*. The filtered posterior distribution of the state, conditional on the observations **y**_0:*t*_, bears a form of *recursive* Bayesian estimation as follows:

(23) p(xt ∣ y0:t)=p(xt)p(y0:t ∣ xt)p(y0:t)=p(xt)p(yt,y0:t−1 ∣ xt)p(yt,y0:t−1)=p(xt)p(yt ∣ xt,y0:t−1)p(y0:t−1 ∣ xt)p(yt ∣ y0:t−1)p(y0:t−1)=p(xt)p(yt ∣ xt,y0:t−1)p(xt ∣ y0:t−1)p(y0:t−1)p(yt ∣ y0:t−1)p(y0:t−1)p(xt)=p(yt ∣ xt,y0:t−1)p(xt ∣ y0:t−1)p(yt ∣ y0:t−1)=p(yt ∣ xt)p(xt ∣ y0:t−1)p(yt ∣ y0:t−1),

where the first four steps are derived from Bayes' rule and the last equality of (23) assumes the conditional independence between the observations. The one-step state prediction, also known as the *Chapman-Kolmogorov equation* [58], is given by

(24) $$\begin{array}{c} p\left(x_{t}\;|\;y_{0:t-1}\right)=\int p\left(x_{t}\;|\;x_{t-1}\right)p\left(x_{t-1}\;|\;y_{0:t-1}\right)dx_{t-1}, \end{array}$$

where the probability distribution (or density) *p*(**x**_*t*_ | **x**_*t*−1_) describes a state transition equation and the probability distribution (or density) *p*(**y**_*t*_ | **x**_*t*_) is the observation equation. Together (23) and (24) provide the fundamental relations to conduct state space analyses. The above formulation of recursive Bayesian estimation holds for both continuous and discrete variables, for either **x** or **y** or both. When the state variable is discrete and countable (in which we use *S*_*t*_ to replace **x**_*t*_), the SSM is also referred to as a hidden Markov model (HMM), with associated *p*(*S*_*t*_ | *S*_*t*−1_) and *p*(**y**_*t*_ | *S*_*t*_). Various approximate Bayesian methods for the HMM have been reported [23, 59, 60]. When the hidden state consists of both continuous and discrete variables, the SSM is referred to as a switching SSM, with associated *p*(**x**_*t*_ | **x**_*t*−1_, *S*_*t*_) and *p*(**y**_*t*_ | **x**_*t*_, *S*_*t*_) [27, 61]. In this case, the inference and prediction involve multiple integrals or summations. For example, the prediction equation (24) will be rewritten as

(25) p(xt ∣ y0:t−1,S0:t−1)=∫∑St−1p(xt ∣ xt−1,St)p(St ∣ St−1)    ×p(xt−1 ∣ y0:t−1,S0:t−1)dxt−1

whose exact or naive implementation can be computationally prohibitive given a large discrete state space.

When the state and observation equations are both continuous and Gaussian, the Bayesian filtering solution yields the celebrated Kalman filter [62], in which the posterior mean and posterior variance are updated recursively. In fact, based on a Gaussian approximation of nonnegative spike count observations, the Kalman filter has been long used in spike train analysis [63, 64]. However, such a naive Gaussian approximation does not consider the point process nature of neural spike trains. Brown and his colleagues [65–67] have proposed a point process filter to recursively estimate the state or parameter in a dynamic fashion. Without loss of generality, assume that the CIF (6) is characterized by a parameter *θ* via an exponential form, namely, *λ*_*t*_ ≡ *λ*(*t* | *θ*_*t*_) = exp⁡(*θ*_*t*_^*⊤*^ *X*_*t*_), and assume that the parameter follows a random-walk equation *θ*_*t*_ = *θ*_*t*−1_ + *w*_*t*_ (where *w*_*t*_ denotes random Gaussian noise with zero mean and variance *σ* ^2^); then one can use a point process filter to estimate the time-varying parameter *θ* at arbitrarily fine temporal resolution (i.e., the bin size can be as small as possible for the discrete-time index *t*) as follows:

(26) $$\begin{array}{c} \theta_{t+1\;|\;t}=\theta_{t\;|\;t}\;\;\left(\text{one-step}\;\text{mean}\;\text{prediction}\right), \end{array}$$

(27) $$\begin{array}{c} V_{t+1\;|\;t}\left(\theta \right)=V_{t+1\;|\;t}\left(\theta \right)+\sigma^{2}\;\;\left(\text{one-step}\;\text{variance}\;\text{prediction}\right), \end{array}$$

(28) θt+1 ∣ t+1=θt+1 ∣ t+Vt+1 ∣ t(θ)∇θλ(θt+1 ∣ t)λ(θt+1 ∣ t) ×[dyt+1−λ(θt+1 ∣ t+1)Δ]=θt+1 ∣ t+Vt+1 ∣ t(θ)Xt+1 ×[dyt+1−λ(θt+1 ∣ t+1)Δ]  (posterior  mode),

(29) Vt+1 ∣ t+1(θ)=[(Vt+1 ∣ t(θ))−1+Xt+1Xt+1⊤λ(θt+1 ∣ t)Δ]−1(posterior  variance),

where *θ*_*t*+1∣*t*+1_ and *V*_*t*+1∣*t*+1_(*θ*) denote the posterior mode and posterior variance for the parameter *θ*, respectively. Equations (26)–(29) are reminiscent of Kalman filtering. Equations (26) and (27) for one-step mean and variance predictions are the same as Kalman filtering, but (28) and (29) are different from Kalman filtering due to the presence of non-Gaussian observations and nonlinear operation in (28). In (28), [*dy*_*t*+1_ − *λ*(*θ*_*t*+1∣*t*+1_)Δ] is viewed as the innovations term, and *V*_*t*+1∣*t*_ *X*_*t*+1_ may be interpreted as a “Kalman gain.” The quantity of the Kalman gain determines the “step size” in error correction. In (29), the posterior state variance is derived by inverting the second derivative of the log-posterior probability density log⁡*p*(*θ*_*t*_ | *Y*) based on a Gaussian approximation of the posterior distribution around the posterior mode [65–67]. For this simple example, we have

(30) log⁡p(θt ∣ Y0:t,Ht)  ∝−12(θt−θt−1 ∣ t−1)⊤Vt+1 ∣ t−1(θt−θt−1 ∣ t−1)     +[log⁡λtdyt−λtΔ],∂log⁡p(θt ∣ Y0:t,Ht)∂θt  =−(θt−θt−1 ∣ t−1)⊤Vt+1 ∣ t−1   +1λt∇θλt[dyt−λtΔ],∂2log⁡p(θt ∣ Y0:t,Ht)∂θt∂θt⊤  =−Vt+1 ∣ t−1+[(∂2λt∂θt∂θt⊤1λt−  (∂λt∂θt)21λt2)×[dyt−λtΔ]−(∂λt∂θt)21λtΔ].

Setting the first-order derivative ∂log⁡*p*(*θ*_*t*_ | *Y*_0:*t*_, *H*_*t*_)/∂*θ*_*t*_ to zero and rearranging terms yield (28), and setting *V*_*t*+1∣*t*+1_(*θ*) = −[∂^2^log⁡*p*(*θ*_*t*_ | *Y*_0:*t*_, *H*_*t*_)/(∂*θ*_*t*_∂*θ*_*t*_^*⊤*^)]^−1^ yields (29).

The Gaussian approximation is based on the first-order Laplace method. In theory one can also use a second-order method to further improve the approximation accuracy [68]. However, in practice the performance gain is relatively small in the presence of noise and model uncertainty while analyzing real experimental data sets. Although the above example only considers a univariate point process (i.e., a single neuronal spike train), it is straightforward to extend the analysis to multivariate point processes (multiple neuronal spike trains). When the number of the neurons increases, the accuracy of Gaussian approximation of log-posterior also improves due to the *Law of large numbers*.

An alternative way for estimating a non-Gaussian posterior is to use a particle filter [69]. Several reports have been published in the context of neural spike train analysis [70, 71]. The basic idea of particle filtering is to employ sequential Monte Carlo (importance sampling and resampling) methods and draw a set of independent and identically distributed (i.i.d.) samples (i.e., “particles”) from a *proposal distribution*; the samples are propagated through the likelihood function, weighted, and reweighted after each iteration update. In the end, one can use Monte Carlo samples (or their importance weights) to represent the posterior. For example, to evaluate the expectation of a function *f*(**x**_*t*_) with respect to the posterior *p*(**x**_*t*_ | **y**_0:*t*_), we have

(31) 𝔼[f(xt)]=∫f(xt)p(xt ∣ y0:t)q(xt ∣ y0:t)q(xt ∣ y0:t)dxt=∫f(xt)W(xt)q(xt ∣ y0:t)dxt≈∑i=1Ncf(xt(i))W(xt(i))∑i=1NcW(xt(i))=f^(xt),

where *W*(**x**_*t*_) = *p*(**x**_*t*_ | **y**_0:*t*_)/*q*(**x**_*t*_ | **y**_0:*t*_) denotes the importance weight function and {**x**_*t*_^(*i*)^}_*i*=1_^*N*_*c*_^ denotes the *N*_*c*_ particles drawn from the proposal distribution *q*(**x**_*t*_ | **y**_0:*t*_). When the sample size *N*_*c*_ is sufficiently large (depending on the dimensionality of **x**), the estimate $\hat{f}(x_{t})$ will be an unbiased estimate of *𝔼*[*f*(**x**_*t*_)]. Based on sequential important sampling (SIS), the importance weights of each sample can be recursively updated as follows [69]:

(32) $$\begin{array}{c} W\left(x_{t}^{\left(i\right)}\right)=W\left(x_{t-1}^{\left(i\right)}\right)\frac{p\left(y_{t}\;|\;x_{t}^{\left(i\right)}\right)p\left(x_{t}^{\left(i\right)}\;|\;x_{t-1}^{\left(i\right)}\right)}{q\left(x_{t}^{\left(i\right)}\;|\;x_{0:t-1}^{\left(i\right)},y_{t}\right)}. \end{array}$$

In practice, choosing a proper proposal distribution *q*(**x**_*t*_ | **x**_0:*t*−1_, **y**_*t*_) is crucial (see [69] for detailed discussions). In the neuroscience literature, Brockwell et al. [70] used a transition prior *p*(**x**_*t*_ | **x**_*t*−1_) as the proposal distribution, which yields a simple form of update from (32) as follows:

(33) $$\begin{array}{c} W\left(x_{t}^{\left(i\right)}\right)=W\left(x_{t-1}^{\left(i\right)}\right)p\left(y_{t}\;|\;x_{t}^{\left(i\right)}\right). \end{array}$$

That is, the importance weights *W*(**x**_*t*_^(*i*)^) are only scaled by the instantaneous likelihood value. Despite its simplicity, the transition prior proposal distribution completely ignores the information of current observation **y**_*t*_. To overcome this limitation, Ergun et al. [71] used a filtered (Gaussian) posterior density derived from the point process filter as the proposal distribution, and they reported a significant performance gain in estimation while maintaining the algorithmic simplicity (i.e., sampling from a Gaussian distribution). In addition, the VB approach can be integrated with particle filtering to obtain a variational Bayesian filtering algorithm [72].

*Note.* (i) If the online operation is not required, we can estimate a smoothed posterior distribution *p*(**x**_*t*_ | **y**_0:*T*_) to obtain a more accurate estimate. The above Bayesian filters can be extended to the fixed-lag Kalman smoother, point process smoother, and particle smoother [63, 66, 69]. (ii) For neural spike train analysis, the formulation of Bayesian filtering is applicable not only to simple point processes but also to marked point processes [73] or even spatiotemporal point processes.

### 3.7. Bayesian Nonparametrics

The contrasting methodological pairs “*frequentist* versus *Bayes*” and “*parametric* versus *nonparametric*” are two examples of dichotomy in modern statistics [74]. The historical roots of Bayesian nonparametrics are dated back to the late 1960s and 1970s. Despite its theoretic development over the past few decades, successful applications of nonparametric Bayesian inference have not been widespread until recently, especially in the field of machine learning [75]. Since nonparametric Bayesian models accommodate a large number of degrees of freedom (infinite-dimensional parameter space) to exhibit a rich class of probabilistic structure, such approaches are very powerful in terms of data representation. The fundamental building blocks are two stochastic processes: Dirichlet process (DP) and Gaussian process (GP). Although detailed technical reviews of these topics are far beyond the scope of this paper, we would like to point out the strengths of these methods in two aspects of statistical data analysis.

- Data clustering, partitioning, and segmentation: unlike the finite mixture models, nonparametric Bayesian models define a prior distribution over the set of all possible partitions, in which the number of clusters or partitions may grow as the increase of the data samples in both static and dynamic settings, including the infinite Gaussian mixture model, Dirichlet process mixtures, Chinese restaurant process, and infinite HMM [74–76]. The model selection issue is resolved implicitly in the process of infinite mixture modeling.
- Prediction and smoothing: unlike the fixed finite-dimensional parametric models, the GP defines priors for the mean function and covariance function, where the covariance kernel function determines the smoothness and stationarity between the data points. Since the predictive posterior is Gaussian, the prediction uncertainty can be computed analytically [28, 77].

Therefore, Bayesian nonparametrics offer greater flexibility for modeling complex data structures. Unfortunately, most inference algorithms for Bayesian nonparametric models involve MCMC methods, which can be computationally prohibitive for large-scale neural data analysis. Therefore, exploiting the sparsity structure of specific neural data and designing efficient inference algorithms are two important directions in practical applications [78].

## 4. Bayesian Methods for Neural Spike Train Analysis

In this section, we review some representative applications of Bayesian methods for neural spike train analysis, with specific emphases on the real experimental data. Nevertheless, the list of the references is by no means complete, and some other complementary references can be found in [79, 80]. Specifically, the strengths of the Bayesian methods are highlighted in comparison with other standard methods; the potentially issues arising from these methods are also discussed.

### 4.1. Spike Sorting and Tuning Curve Estimation

To characterize the firing property of single neurons, it is necessary to first *identify* and *sort* the spikes from the recorded multiunit activity (MUA) (which is referred to as the discrete ensemble spikes passing the threshold criterion) [81–83]. However, spike sorting is often a difficult and error-prone process. Traditionally, spike sorting is formulated as a clustering problem based on spike waveform features [84]. Parametric and nonparametric Bayesian inference methods have been developed for mixture modeling and inference (e.g., [25, 26]), especially for determining the model size [85, 86]. Unlike the maximum likelihood estimation (which produces a hard label for each identified spike), Bayesian approaches produce a soft label (posterior probability) for individual spike; such uncertainties may be considered in subsequent analyses (such as tuning curve estimation and decoding). Spike sorting can also be formulated as a dynamic model inference problem, in the context of state space analysis [87] or in the presence of nonstationarity [88]. Recent studies have suggested that spike sorting should take into account not only spike waveform features but also the neuronal tuning property [89, 90], suggesting that these two processes shall be integrated.

At the single neuron level, a Poisson neuronal firing response is completely characterized by its tuning curve or receptive field (RF). Naturally, estimating the neuronal tuning curve is the second step following spike sorting. Standard tuning curve or RF estimation methods include the spike-triggered average (STA) and spike-triggered covariance (STC). The Bayesian versions of the STA and STC have been proposed [91, 92]. Binning and smoothing are two important issues in firing rate estimation Bayesian methods provide a principled way to estimate the peristimulus time histogram (PSTH) [93]. For estimating a time-varying firing rate profile similar to PSTH, the Bayesian adaptive regression splines (BARS) method offers a principled solution for bin size selection and smoothing based on the RJ-MCMC method [94]. Notably, BARS is more computationally intensive. For similar estimation performance (validated on simulated data), a more computationally efficient approach has been developed using Bayesian filtering-based state space analysis [95]. In addition, Metropolis-type MCMC approaches have been proposed for high-dimensional tuning curve estimation [96, 97].

### 4.2. Neural Encoding and Decoding

The goal of neural encoding is to establish a statistical mapping (which can be either a biophysical or data-driven model) between the stimulus input and neuronal responses, and the goal of neural decoding is to extract or reconstruct information of the stimulus given the observed neural signals. For instance, the encoded and decoded variables of interest can be a rodent's position during spatial navigation, the monkey's movement kinematics in a reach-to-grasp task, or specific visual/auditory/olfactory stimuli during neuroscience experiments.

Without loss of generality, let $\left\{\tilde{X},\tilde{Y}\right\}$ denote the *observed* stimuli and neuronal responses, respectively, at the encoding stage, and let *θ* denote the model parameter of a specific encoding model *ℳ*; then the posterior distribution of the model (and model parameters) is written as

(34) $$\begin{array}{c} p\left(\theta ,ℳ\;|\;\tilde{X},\tilde{Y}\right)\propto p\left(\tilde{X},\tilde{Y}\;|\;\theta ,ℳ\right)p\left(\theta \;|\;ℳ\right)p\left(ℳ\right). \end{array}$$

Once the model *ℳ* is determined, one can infer the posterior mean by $\hat{\theta }=\int \theta p(\theta |\tilde{X},\tilde{Y},ℳ)d\theta$. Depending on the selected likelihood or prior, variations of Bayesian neural encoding methods have been developed [98–100].

Given the parameter posterior $p(\theta |\tilde{X},\tilde{Y},ℳ)$ from the encoding analysis, decoding analysis aims to infer the latent variable *X* given new data *Y* at the decoding stage (with preselected *ℳ*). Within the Bayesian framework, it is equivalent to finding the *X*_MAP_ [101] as follows:

(35) XMAP=argmax⁡X⁡p(X ∣ θ,Y,ℳ)=argmax⁡X⁡∫p(Y ∣ X,θ,ℳ)p(θ ∣ X~,Y~,ℳ)p(X)dθ≈argmax⁡X⁡p(Y ∣ X,θ^,ℳ)p(X),

which consists of two numerical problems: *maximization* and *integration*. In the approximation in the last step of (35), we have used $p(\theta |\tilde{X},\tilde{Y},ℳ)\approx \delta (\theta -\hat{\theta })$, where $\hat{\theta }$ denotes the estimated mean or mode statistic from $p(\theta |\tilde{X},\tilde{Y},ℳ)$. The optimization problem is more conveniently written in the log domain as follows:

(36) $$\begin{array}{c} \log p\left(X\;|\;Y,\hat{\theta }\right)\propto \log p\left(Y\;|\;X,\hat{\theta }\right)+\log p\left(X\right). \end{array}$$

If *X* follows a Markovian process, this can be solved by recursive Bayesian filtering [65, 67] (Section 3.6). When *X* is non-Markovian but *p*(*X*) and the likelihood are both log-concave, this can be resorted to a global optimization problem [57, 102]. Imposing prior information and structure (e.g., sparsity, spatiotemporal correlation) onto *p*(*X*) is important for obtaining either a meaningful solution or a significant optimization speedup [103, 104]. In contrast, when *p*(*X*) is flat or noninformative, the MAP solution will be similar to the m.l.e.

In the literature, the majority of neural encoding or decoding models fall within two parametric families: linear model (e.g., [63, 105]) and GLM (e.g., [64, 106, 107]), although nonparametric encoding models have also been considered [108, 109]. Methods for Bayesian neural decoding include (i) Kalman filtering [63], (ii) point process filtering [65–67, 110, 111], (iii) particle filtering [70, 71], and (iv) MCMC methods [112]. The areas of experimental neuroscience data include the retina, primary visual cortex, primary somatosensory cortex, auditory periphery (auditory nerves and midbrain auditory neurons), primary auditory cortex, primary motor cortex, premotor cortex, hippocampus, and the olfactory bulb.

It is important to point out that most spike-count or point process based decoding algorithms rely on the assumptions that neural spikes have been properly sorted (some neural decoding algorithms (e.g., [113]) are based on detected MUA instead of sorted single unit activity). Recently, there have been a few efforts in developing spike-sorting-free decoding algorithms, by either estimating the cell identities as missing variables [114] or modeling the spike identities by their proxy based on a spatiotemporal point process [115, 116]. Although this work has been carried out using likelihood inference, it is straightforward to extend it to the Bayesian framework. In the example of decoding the rat's position from recorded ensemble hippocampal spike activity [115, 116], we used a model-free (without *θ*) and data-driven Bayes' rule as follows:

(37) $$\begin{array}{c} p\left(X\;|\;Y,\tilde{X},\tilde{Y}\right)\propto p\left(Y\;|\;X,\tilde{X},\tilde{Y}\right)p\left(X\right), \end{array}$$

in which *p*(*X*) denotes the prior and the likelihood $p(Y|X,\tilde{X},\tilde{Y})$ is evaluated nonparametrically (namely, nonparametric neural decoding). By assuming that the joint/marginal/conditional distributions (*p*(*X*, *Y*) and $p(\tilde{X},\tilde{Y})$, *p*(*X*) and $p(\tilde{X})$, and *p*(*Y* | *X*) and $p(\tilde{Y}|\tilde{X})$) are stationary during both encoding and decoding phases, the MAP estimate of decoding analysis is obtained by

(38) XMAP =argX⁡max⁡⁡p(Y ∣ X,X~,Y~)p(X) ≈argX⁡max⁡⁡f(Y|p(X ∣ X~),p(X,Y ∣ X~,Y~))p(X),

where *f* is a nonlinear function that involves the marginal and joint pdf's in the argument [115, 116], in which the pdf's are constructed based on a kernel density estimator (KDE). Alternatively, the nonparametric pdf in (38) can be replaced by a parametric form [115] as follows:

(39) $$\begin{array}{c} X_{\text{MAP}}\approx \underset{X}{\arg}\max f\left(Y|p\left(X\;|\;\theta \right),p\left(X,Y\;|\;\theta \right)\right)p\left(X\right), \end{array}$$

where *p*(*X* | *θ*) = ∫*p*(*X*, *Y* | *θ*)*dY* is the parametric marginal and *θ* is the point estimate obtained from the training samples $\left\{\tilde{X},\tilde{Y}\right\}$.

*Note.* (i) Neural encoding and decoding analyses are established upon the assumption that the neural codes are well understood—namely, how neuronal spikes represent and transmit the information of the external world. Whether being a rate code, a timing code, a latency code, or an independent or correlated population code, Bayesian approach provides a universal strategy to test the coding hypothesis or extract the information [117]. (ii) The sensitivity of spike trains to noise may affect the effectiveness to the encoding-decoding process. From an information-theoretic perspective, various sources of spike noise, such as misclassified spikes (false positives) and misdetected, or misclassified spikes (false negatives), may affect differently the mutual information between the input (stimulus) and output (spikes) channel [118, 119]. In designing a Bayesian decoder, it is important to take into account the noise issue. A decoding strategy that is robust to the noise assumption will presumably yield the best performance [115, 116].

### 4.3. Deconvolution of Neural Spike Trains

Fluorescent calcium imaging tools have become increasingly popular for observing the spiking activity of large neuronal populations. However, extracting or deconvolving neural spike trains from the raw fluorescence movie or video sequences remains a challenging estimation problem. The standard *dF*/*F* or Wiener filtering approaches do not capture the true statistics of neural spike trains and are sensitive to the noise statistics [120].

A principled approach is to formulate the deconvolution problem of a filtered point process via state space analysis and Bayesian inference [121, 122] (see also [123] for another type of Bayesian deconvolution approach using MCMC). Let *F*_*t*_ denote the measured univariate fluorescence time series, which is modeled as a linear Gaussian function of the intracellular calcium concentration ([Ca^2+^]) as follows:

(40) $$\begin{array}{c} F_{t}=\alpha {\left[\text{C}{\text{a}}^{2+}\right]}_{t}+\beta +\epsilon_{t}, \end{array}$$

where *β* denotes a constant baseline and *ϵ*_*t*_ ~ *𝒩*(0, *σ* ^2^) denotes the Gaussian noise with zero mean and variance *σ* ^2^. The calcium concentration can be modeled as a first-order autoregressive (AR) process corrupted by Poisson noise as follows:

(41) $$\begin{array}{c} \alpha {\left[\text{C}{\text{a}}^{2+}\right]}_{t}=\alpha {\left[\text{C}{\text{a}}^{2+}\right]}_{t-1}+n_{t}, \end{array}$$

where $n_{t}\sim Poisson\;(\lambda \Delta )$ and the bin size Δ is chosen to assure that the mean firing rate is independent of the imaging frame rate.

Let *θ* = {*α*, *β*, *γ*, *σ* ^2^, *λ*}; given the above generative biophysical model, Bayesian deconvolution aims to seek the MAP estimate of spike train as follows:

(42) n^=argmax⁡nt∈ℕ0⁡ p(n ∣ F,θ)=argmax⁡nt∈ℕ0⁡ p(F ∣ n,θ)p(n ∣ θ)=argmax⁡nt∈ℕ0⁡∏t=1Tp(Ft ∣ Cat2+,θ)∏t=1Tp(nt ∣ θ).

Within the state space framework, Vogelstein and colleagues [121] proposed a particle filtering method to infer the posterior probability of spikes at each imaging frame, given the entire fluorescence traces. However, the Monte Carlo approach is computationally expensive and may not be suitable for analyses of a large population of neurons. To meet the real-time processing requirement, they further proposed an approximate yet fast solution by replacing the Poisson distribution by an exponential distribution with the same mean (therefore relaxing the nonnegative integer constraint to the nonnegative real number) [122]. And the approximate solution is given by the following optimization problem:

(43) n^=argmax⁡nt≥0⁡ ∑t=1T−12σ2(Ft−αCat2+−β)2−ntλΔ=argmax⁡Cat2+−γCat−12+≥0⁡ ∑t=1T−12σ2(Ft−αCat2+−β)2−(Cat2+−γCat−12+)λΔ.

The approximation of exponential form makes the optimization problem concave with respect to Ca^2+^, from which the global optimum can be obtained using constrained convex optimization [102]. Once the estimate of the calcium trace is obtained, the MAP spike train can be simply inferred by a linear transformation.

In a parallel fashion, the parameter *θ* can be similarly estimated by Bayesian inference as follows:

(44) θMAP=argmax⁡θ⁡∫p(F ∣ Ca2+,θ)p(Ca2+ ∣ θ)dCa2+≈argmax⁡θ⁡p(F ∣ n^,θ)p(n^ ∣ θ),

where the approximation in the second step assumes that the major mass in the integral is around the MAP sequence $\hat{n}$ (or equivalently the Ca^2+^ traces). Therefore, the joint estimate $\left(\hat{n},\theta_{\text{MAP}}\right)$ can be computed iteratively from (43) and (44) until convergence.

*Note.* The output of Bayesian deconvolution yields a probability vector between 0 and 1 of having a spike in a given time frame. Selection of different thresholds on the probability vector leads to different detection errors (a tradeoff between the false positives and false negatives). Nevertheless, the Bayesian solution is much more superior to the standard least-squares method. It is noteworthy that a new fast deconvolution method has recently been proposed based on finite rate of innovation (FRI) theory, with reported performance better than the approximate Bayesian solution [124].

### 4.4. Inference of Neuronal Functional Connectivity and Synchrony

Identifying the functional connectivity of simultaneously recorded neuronal ensembles is an important research objective in computational neuroscience. This analysis has many functional applications such as in neural decoding [125] and in understanding the collective dynamics of coordinated spiking cortical networks [126]. Compared to the standard nonparametric approaches such as cross-correlogram and joint peristimulus time histogram (JPSTH), parametric model-based statistical approaches offer several advantages in neural data interpretation [127].

To model the spike train point process data, without loss of generality we use the following logistic regression model with a logit link function. Specifically, let *c* be the index of a target neuron, and let *i* = 1,…*C* be the indices of triggered neurons (whose spike activity is assumed to trigger the firing of the target neuron). The Bernoulli (binomial) logistic regression GLM is written as

(45) logit(πt)=θc⊤Xt=θ0c+∑j=1Jθjcxj,t=θ0c+∑i=1C ∑k=1Kθi,kcxi,t−k,

where dim⁡(*θ*_*c*_) = *J* + 1 = *C* × *K* + 1 for the augmented parameter vector *θ*_*c*_ = {*θ*_0_^*c*^, *θ*_*i*,*k*_^*c*^} and *X*_*t*_ = {*x*_0_, *x*_*i*,*t*−*k*_}. Here, *x*_0_ ≡ 1, and *x*_*i*,*t*−*k*_ denotes the raw spike count from neuron *i* at the *k*th time-lag history window (or a predefined smooth basis function such as in [125]). The spike count is nonnegative; therefore *x*_*i*,*t*−*k*_ ≥ 0. Alternatively, (45) can be rewritten as

(46) $$\begin{array}{c} \pi_{t}=\frac{\exp\left(\theta_{c}^{⊤}X_{t}\right)}{1+\exp\left(\theta_{c}^{⊤}X_{t}\right)}=\frac{\exp\left(\theta_{0}^{c}+\sum_{j=1}^{J}\theta_{j}^{c}x_{j,t}\right)}{1+\exp\left(\theta_{0}^{c}+\sum_{j=1}^{J}\theta_{j}^{c}x_{j,t}\right)}, \end{array}$$

which yields the probability of a spiking event at time *t*. Equation (46) defines a spiking probability model for neuron *c* based on its own spiking history and that of the other neurons in the ensemble. Here, exp⁡(*θ*_0_^*c*^) can be interpreted as the baseline firing probability of neuron *c*. Depending on the algebraic (positive or negative) sign of coefficient *θ*_*i*,*k*_^*c*^, exp⁡(*θ*_*i*,*k*_^*c*^) can be viewed as a “gain” factor (dimensionless, >1 or <1) that influences the relative firing probability of neuron *c* from another neuron *i* at the previous *k*th time lag. Therefore, a negative value of *θ*_*i*,*k*_^*c*^ will strengthen the inhibitory effect; a positive value of *θ*_*i*,*k*_^*c*^ will enhance the excitatory effect. Two neurons are said to be functionally connected if any of their pairwise connections is nonzero (or the statistical estimate is significantly nonzero).

For inferring the functional connectivity of neural ensembles, in addition to the standard likelihood approaches [127, 128], various forms of Bayesian inference have been developed for the MaxEnt model, GLM, and Bayesian network [129–132]. In a similar context, a Bayesian method has been developed based on the deconvolved neuronal spike trains from calcium imaging data [133].

Bayesian methods also proved useful in detecting higher-order correlations among neural assemblies [134, 135]. Higher-order correlations are often characterized by synchronous neuronal firing at a timescale of 5–10 ms. These findings have been reported in experimental data from prefrontal cortex, somatosensory cortex, and visual cortex across many species and animals. Consider a set of *C* neurons. Each neuron is represented by two states: 1 (firing) or 0 (silent). At any time instant, the state of the *C* neurons is represented by the vector *X* = (*x*_1_, *x*_2_,…, *x*_*C*_) (the time index is omitted for simplicity), and in total there are 2^*C*^ neuronal states. For instance, a general joint distribution of three neurons can be expressed by a log-linear model [134]

(47) p(x1,x2,x3)=exp⁡(θ0+θ1x1+θ2x2+θ3x3+θ12x1x2+θ13x1x3+θ23x2x3+θ123x1x2x3),

which is a natural extension of the MaxEnt model described in (9). A nonzero coefficient of *θ*_123_ would imply the presence of third-order correlation among the three neurons. In experimental data, the number of synchronous events may be scarce in single trials, and the interaction coefficients may also be time-varying. State space analysis and Bayesian filtering offer a principled framework to address these issues [135]. However, the computational bottleneck is the curse of dimensionality when the value of *C* is moderately large (2^20^ ≈ 10^6^). In the presence of finite data sample size, it is reasonable to impose certain structural priors onto the parameter space for the Bayesian solution.

## 5. Discussion

We have presented an overview of Bayesian inference methods and their applications to neural spike train analysis. Although the focus of current paper is on neural spike trains, the Bayesian principle is also applicable to other modalities of neural data (e.g., [136]). Due to space limitation, we only cover representative methods and applications in this paper, and the references are reflective of our personal choices from the humongous literature.

In comparison with the standard methods, Bayesian methods provide a flexible framework to address many fundamental estimation problems at different stages of neural data analysis. Regardless of the specific Bayesian approach to be employed, the common goal of Bayesian solutions consists in replacing a single point estimate (or hard decision label) with a full posterior distribution (or soft decision label). As a tradeoff, Bayesian practioners have to encounter the increasing cost of computational complexity (especially while using MCMC), which may be prohibitive for large-scale spike train data sets. Furthermore, special attention shall be paid to select the optimal technique among different Bayesian methods that ultimately lead to quantitatively different approximate Bayesian solutions.

Despite the significant progresses made to date, there remain many research challenges and opportunities for applying Bayesian machinery to neural spike trains, and we will mention a few of them below.

### 5.1. Nonstationarity

Neural spiking activity is highly nonstationary at various timescales. Sources that account for such nonstationarity may include the animal's behavioral variability across trials, top-down attention, learning, motivation, or emotional effects across time. These effects are time-varying across behaviors. In addition, individual neuronal firing may be affected by other unobserved neural activity, such as through modulatory or presynaptic inputs from other nonrecorded neurons. Therefore, it may be important to consider these latent variables while analyzing neural spike trains [137]. Bayesian methods are a natural solution to model and infer such latent variables. Traditional mixed-effects models can be adapted to a hierarchical Bayesian model to capture various sources of randomness.

### 5.2. Characterization of Neuronal Dependencies

Neural responses may appear correlated or synchronous at different timescales. It is important to characterize such neuronal dependencies in order to fully understand the nature of neural codes. It is also equally important to associate the neural responses to other measurements, such as behavioral responses, learning performance, or local field potentials. Commonly, correlation statistics or information-theoretic measures have been used (e.g., [138]). Other advanced statistical measures have also been proposed, such as the log-linear model [139], Granger causality [140], transfer entropy [141], or copula model [142]. Specifically, the copula offers a universal framework to model statistical dependencies among continuous, discrete, or mixed-valued r.v., and it has an intrinsic link to the mutual information; Bayesian methods may prove useful for selecting the copula class or the copula mixtures [143]. However, because of the nonstationary nature of neural codes (Section 5.1), it remains a challenge to identify the “true” dependencies among the observed neural spike trains, and it remains important to rule out and rule in neural codes under specific conditions.

### 5.3. Characterization and Abstraction of Neuronal Ensemble Representation

Since individual neuronal spike activity is known to be stochastic and noisy, in the single-trial analysis it is anticipated that the information extracted from neuronal populations is more robust than that from a single neuron. How to uncover the neural representation of population codes in a single-trial analysis has been an active research topic in neuroscience. This is important not only for abstraction, interpretation, and visualization of population codes but also for discovering invariant neural representations and their links to behavior. Standard dimensionality reduction techniques (e.g., principle component analysis, multidimensional scaling, or locally linear embedding) have been widely used for such analyses. However, these methods have ignored the temporal component of neural codes. In addition, no explicit behavioral correlate may become available in certain modeling tasks. Recently, Bayesian dynamic models, such as the Gaussian process factor analysis (GPFA) [144] and VB-HMM [145–147], have been proposed to visualize population codes recorded from large neural ensembles across different experimental conditions. To learn the highly complex structure of spatiotemporal neural population codes, it may be beneficial to borrow the ideas from the machine learning community and to integrate the state-of-the-art unsupervised and supervised deep Bayesian learning techniques.

### 5.4. Translational Neuroscience Applications

Finally in the long run, it is crucial to apply basic neuroscience knowledge derived from quantitative analyses of neural data to translational neuroscience research. Many clinical research areas may benefit from the statistical analyses reviewed here, such as design of neural prosthetics for patients with tetraplegia [107], detection and control of epileptic seizures, optical control of neuronal firing in behaving animals, or simulation of neural firing patterns to achieve optimal electrotherapeutic effect [148]. Bridging the gap between neural data analysis and their translational applications (such as treating neurological or neuropsychiatric disorders) would continue to be a prominent mission accompanying the journey of scientific discovery.

## Figures and Tables

**Table 1:** Probability distributions for modeling neuronal spike count observations.

Distribution	Mean statistic	Variance	Note for observations *Y*
Binomial (*p*)	*𝔼*[*Y*] = *p*	*p*(1 − *p*)	*Y* ∈ {0,1}
Poisson (*λ*)	*𝔼*[*Y*] = *λ*	*λ*	*Y* ≥ 0, *Y* ∈ *ℤ* ^+^
Negative binomial (*r*, *p*)	*𝔼*[*Y*] = *pr*/(1 − *p*)	*pr*/(1 − *p*)^2^	*Y* ≥ 0, *Y* ∈ *ℤ* ^+^ (overdispersed Poisson)
Skellam (*μ*_1_, *μ*_2_)	*𝔼*[*Y*] = *μ*_1_ − *μ*_2_	*μ*_1_ + *μ*_2_	*Y* ∈ *ℤ* (difference between two Poissons)
